# Multifaceted Health Benefits of *Mangifera indica* L. (Mango): The Inestimable Value of Orchards Recently Planted in Sicilian Rural Areas

**DOI:** 10.3390/nu9050525

**Published:** 2017-05-20

**Authors:** Marianna Lauricella, Sonia Emanuele, Giuseppe Calvaruso, Michela Giuliano, Antonella D’Anneo

**Affiliations:** 1 Department of Experimental Biomedicine and Clinical Neurosciences, Laboratory of Biochemistry, University of Palermo, via del Vespro 129, 90127 Palermo, Italy; marianna.lauricella@unipa.it (M.L.); sonia.emanuele@unipa.it (S.E.); 2 Department of Biological, Chemical and Pharmaceutical Sciences and Technologies, Laboratory of Biochemistry, University of Palermo, via del Vespro 129, 90127 Palermo, Italy; giuseppe.calvaruso@unipa.it (G.C.); michela.giuliano@unipa.it (M.G.)

**Keywords:** *Mangifera indica* L. fruit, nutraceutical properties

## Abstract

Historically, *Mangifera indica* L. cultivations have been widely planted in tropical areas of India, Africa, Asia, and Central America. However, at least 20 years ago its spreading allowed the development of some cultivars in Sicily, an island to the south of Italy, where the favourable subtropical climate and adapted soils represent the perfect field to create new sources of production for the Sicilian agricultural supply chain. Currently, cultivations of Kensington Pride, Keitt, Glenn, Maya, and Tommy Atkins varieties are active in Sicily and their products meet the requirements of local and European markets. Mango plants produce fleshy stone fruits rich in phytochemicals with an undisputed nutritional value for its high content of polyphenolics and vitamins. This review provides an overview of the antioxidant, anti-inflammatory, and anticancer properties of mango, a fruit that should be included in everyone’s diet for its multifaceted biochemical actions and health-enhancing properties.

## 1. Introduction

Nowadays, the use of medicinal plants and bioactive phytocompounds has seen more growing interest. The importance of a diet rich in polyphenols has long been sponsored and underlined because of their radical scavenging action, as well as anti-carcinogenetic properties [1]. Fruit and vegetables are rich sources of many different bioactive phytocompounds, including phenolic components, anthocyanins, carotenoids, vitamin E, and vitamin C, which exhibit good antioxidant properties and are, therefore, regarded as an unquestionable component that should be present in everyone’s diet [1]. Epidemiologic studies have consistently shown that consumption of fruits and vegetables reduces the incidence of chronic diseases, such as cancer, diabetes, and cardiovascular disease [2,3,4].

Fruits of tropical and subtropical regions are appreciated for their nutritional value, as well as for the presence of health-enhancing compounds. *Mangifera indica* L. (mango) is known as “the king of fruits” because it is the most popular fruit in tropical regions. It is the national fruit of India and the Philippines, and the national tree of Bangladesh [5]. The mango belongs to genus *Mangifera*, which consists of numerous species of tropical fruits in the family of Anacardiaceae [6]. *Mangifera indica* L. is native to India and Southeast Asia [7] where it has been cultivated for over 4000 years for the good qualities of the fruits. Currently, mango is also grown in Central America [8], Africa [9], Australia [10], and for a few years in Europe [11]. Over one thousand mango fruit varieties are available worldwide, although only a few are produced on a commercial scale. In the Mediterranean area, Spain and Israel are the major producing countries. In the south of Italy, particularly in Sicily, mango began to have an initial controversial departure in early 1980s with the introduction of the Kensington Pride cultivar in Catania province. However, it was only 20 years later (2000), after in-depth research, that the different mango cultivars (Kensington Pride, Tommy Atkins, Osteen, Maya, Kent, Irwin, Glenn, and Keitt varieties) spread through Sicily, mainly in the provinces of Palermo, Messina, and Catania, that offer a particularly suitable environment [12,13]. Albeit the spread of local mango orchards can be compromised by pathogenicity [12], this new agricultural recovery of the plant met the farmer’s enthusiasm to cover abandoned soils, previously dedicated to citrus groves and no longer profitable for Sicilian rural market. An added value was also provided by the market surveys showing a growing interest of local, national, and European consumers towards a product that seems to have qualitative and nutraceutical properties similar to those imported by tropical areas, with the advantage of ripening on the tree and offering a fresh fruit.

Our purpose in this review was to provide an exhaustive overview of the main chemical bioactive components and the relevant pharmacological activities (anti-oxidant, anti-inflammatory, and anti-cancer) of the different mango fruit fractions. Throughout the manuscript we also address all pieces of information collected so far on Sicilian mango, underpinning its potential benefits.

## 2. Botanical Characterisation

**Figure 1:**
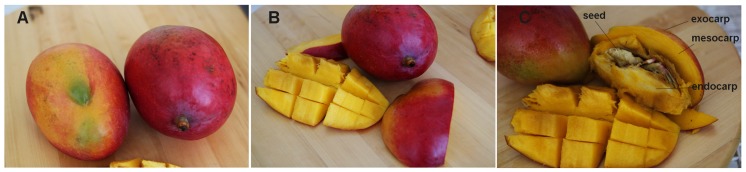
Features of the different mango fruit fractions. The fruits with a thin and colourful peel (**A**); and a yellowish-orange edible flesh (**B**) are shown; (**C**) Details of different mango fractions are reported, illustrating the outer peel (exocarp), the edible pulp (mesocarp) and the stony pit (endocarp) enclosing a large seed inside.

The mango tree is an evergreen, fast-growing, and long-lived orchard. It is very vigorous with a large canopy and an almost circular projection. The leaves are perennial, deep green, pointed, and shiny, while the inflorescence occurs in panicles consisting of about 3000 whitish-red or yellowish–green flowers. In tropical regions the trees can reach up to 30–40 m in height, while in subtropical areas the growth rate is consistently reduced. The mango fruit has hundreds of varieties, each having its own characteristic taste, shape, and size. Each fruit is 5–15 cm long and 4–10 cm in diameter. Usually its weight ranges from 150 g to around 750 g, reaching approximately 390 g in Sicilian mango fruit [14]. The outer peel (exocarp) is smooth and is green in unripe mango, but it turns golden yellow, crimson red, yellow, or orange-red in ripe fruits, depending upon the cultivar type. The endocarp is a large ovoid-oblong core that contains a single seed. The pulp (mesocarp) is orange-yellow in colour, well-endowed with numerous soft fibrils (Figure 1). Its flavour is pleasant and rich, and its taste is sweet with mild tartness. Mango is consumed fresh or is processed for chutney, pickles, curries, dried products, puree, nectar, and canned or frozen slices that are popular worldwide.

The growing interest for *Mangifera indica* L., which has been the focus of attention of many researchers around the tropical and subtropical areas, has to be searched for its phytochemical content that qualified mango fruit as a superfruit model [15].

The chemical analysis of mango pulp provided evidence that it has a relatively high content in calories (60 Kcal/100 g fresh weight) and is an important source of potassium, fibre, and vitamins. The nutritive value of mango is listed in Table 1 reporting some data from the National Nutrient Database for Standard Reference (United States Department of Agriculture) [16].

Mango is also a particularly rich source of polyphenols, a diverse group of organic micronutrients found in plants which exert specific health benefits [17]. Polyphenols identified in mango mesocarp include mangiferin, gallic acid, gallotannins, quercetin, isoquercetin, ellagic acid, and β-glucogallin [18,19], with gallic acid being the most represented phenol compound in this fraction [20]. Furthermore, up to 25 diverse carotenoids have been identified in the mesocarp fraction, such as provitamin A, lutein, α-carotene, and β-carotene that account for the yellowish colour of this part of the fruit.

During the processing of mango, exocarp and seed are discarded. However, several studies report that these mango by-products also contain high levels of health-enhancing compounds. Mango exocarp has been found to be a good source of polyphenols, carotenoids, dietary fibre, and vitamin E [21]. Polyphenols present in mango exocarp include mangiferin, quercetin, rhamnentin, ellagic acid, and kaempferol [18,19]. The analysis of exocarp polyphenolic content of different mango cultivars unveiled the highest level in Josè, Tommy Atkins, Ngowe, Haden, and Heidi varieties [19]. Generally, a higher total polyphenol amount was found in the exocarp of ripe fruits than the unripe ones. [22]. Like mango mesocarp and exocarp, mango seed kernels are also equally rich in polyphenols with potent antioxidant activity. As reported by Jahurul et al. [21] mango seed kernels contain tannin, gallic acid, coumarin, caffeic acid, vanillin, mangiferin, ferulic acid, and cinnamic acid. Finally, polyphenols are also present in mango leaves, flowers, and stem bark.

In traditional medicine the different parts of the mango tree (fruit pulp, extracts of fruit kernel, leaves, and stem bark) are used for their health properties [23]. Decoction of mango kernel is used, for example, in the treatment of diarrhea, haemorrhages, and bleeding haemorrhoids for its vermifuge and astringent properties [24], extracts of unripe fruit, bark and leaves are used for their antibiotic activity [25,26], while an aqueous stem bark extract from *Mangifera indica* L. is used in Cuba as a remedy for diarrhoea, fever, gastritis, and ulcers [27].

Preliminary studies performed on Sicilian mango cultivars indicate that the composition of fruit phytocompounds reflects that found in the fruit cultivated in tropical areas. However, a better analysis must be performed to correlate the total polyphenols (0.53 mg/g gallic acid equivalents) and total flavonoid index (0.85 mg/g gallic acid equivalents) found in Sicilian Kensington Pride to the other tropical cultivars [14], also in relation to the ripeness status of the fruits.

## 3. Antioxidant Properties of *Mangifera indica* L.

Nowadays, particular attention is paid to nutrients capable of counteracting oxidative stress. A certain number of reactive oxygen species, or ROS, including superoxide anions, hydroxyls, and hydrogen peroxide, are produced in the human body. Some of them, such as superoxide anions and hydrogen peroxide, are physiologically generated during the electron transfer in the mitochondrial respiratory chain. Other species, as the hydroxyl radical, one of the more dangerous ROS, is produced by the Fenton reaction, causing the oxidation of Fe^2+^ to Fe^3+^. These derivatives of oxygen, highly unstable and particularly reactive, oxidize atoms or organic molecules, especially cell components such as proteins, lipids, and nucleic acids.

Cells have developed a specific group of enzymatic systems (catalase, superoxide dismutase, glutathione peroxidase, etc.) to remove ROS and many of them are transcriptionally regulated by Nrf2 (nuclear factor erythroid 2-related factor 2)/Keap-1 (Kelch-like ECH-associated protein 1) axis, the master regulatory pathway of the antioxidant response [28]. Under stress conditions, Nrf2 is stabilized and allows survival and stress adaptation, upregulating the expression of some cytoprotective molecules, the antioxidant responses, and the stress-mediated detoxification enzymes (NAD(P)H quinina reductase, glutathione S-transferase, superoxide dismutase, heme oxygenase, catalase, and glutathione peroxidase) [29]. If ROS are not removed, their accumulation overcomes the cellular reparative abilities, causing the collapse of cellular functions and can result in the generation of pathological states related to aging, cancer, atherosclerosis, heart attack, stroke, and diabetes.

It is well known that phytochemical compounds of a phenolic nature commonly found in fruits display free radical scavenging activities, due to the reactivity of the phenol moiety and via hydrogen or electron donation. The large variety of antioxidants, pigments, and vitamins that are present in any part of the mango plant are responsible for the antioxidant and free radical scavenging activities.

The analysis of different commercially-ripe mango varieties from Bangladesh, such as Fazli, Langra, Ashwina, Himsagor, and Amrupali, demonstrated, for example, the existence of differences in functional factors and antioxidant constituents (ascorbic acid and total phenol contents) present in the mesocarp which change from one cultivar to another [30]. Among all analysed cultivars, Langra was found to have the highest phenol content as well as antioxidant properties compared to those of other four mango varieties, whereas Ashwina variety showed the highest content in ascorbic acid. The anti-scavenging activity of these cultivars was positively correlated with both ascorbic acid and total polyphenol contents [30], albeit the authors did not explore the biochemical mechanism responsible for the observed effects. In this scenario, we recently started to explore the antioxidant and anti-aging properties of Sicilian mango extracts exploiting our knowledge in the study of oxidative stress and cell death induction in tumour systems [31,32,33,34,35,36,37]. Using fruit extract from the Kensington Pride cultivar grown and widely spread in Balestrate (Palermo) and other Sicilian rural areas, our preliminary results provided evidence that exocarp, mesocarp, and endocarp of mango can efficaciously counteract oxidative damage caused by ROS. In addition, our data uncovered the role of the enzyme based scavenger systems such as catalase, superoxide dismutase, and their regulator Nrf2 in modulating the anti-oxidant response of Sicilian mango extracts (unpublished data).

## 4. Anti-Inflammatory Effects of Mango

Several studies showed that phytochemicals contained in mango play an anti-inflammatory role in several chronic pathological disorders associated with inflammatory responses [38,39]. Inflammatory bowel diseases, primarily including ulcerative colitis, are disorders that are characterised by chronic inflammation and mucosal damage in the large intestine. This is associated with an increased risk of colon and rectal cancers [40]. Although the exact aetiology of this disease is not fully known, the mucosa of patients has been shown to produce large quantities of pro-inflammatory cytokines, such as IL-1, IL-6, IL-12, and TNF-α [41,42]. These, in turn, induce the expression of enzymes associated with inflammation, such as iNOS and COX-2. The expression of pro-inflammatory cytokines is regulated by the nuclear factor kappa-B (NF-κB), a transcriptional factor whose level has been found increased in the mucosa of inflammatory bowel disease patients [43]. Mango extracts have been shown to exert an anti-inflammatory activity in experimental murine models of ulcerative colitis [44]. The treatment with an aqueous stem bark extract from *Mangifera indica,* containing a mixture of polyphenols and flavonoids, attenuated the colitis symptoms, like body weight loss, colon shortening, and diarrhoea [44]. Moreover, mango extracts reduced the levels of iNOS, COX-2, TNF-α, and TNFR-2 expression in colonic tissue, as well as decreased IL-6 and TNF-α serum levels [44]. These effects can be related to the ability of mango stem bark extract to inhibit NF-κB [45]. Furthermore, Kim et al. [46] reported that mango beverage, a mango mesocarp extract rich in polyphenols (475.90 mg/L gallic acid equivalent), reduces the inflammatory response associated with dextran sodium sulfate-induced colitis in mice by inhibiting the IGF1R/AKT/mTOR pathway. Such an effect was attributed to gallic acid, the most prevalent polyphenol of mango mesocarp, which showed in silico modelling the ability to bind and inhibit the catalytic domain of IGF-1R [21]. In another study the same authors also showed that the inhibition of mTOR pathway by mango polyphenols is in part due to the increased expression of miR-126, an inhibitor of the phosphatidylinositol 3-kinase (PI3K), an upstream activator of mTOR [47]. A large amount of evidence supports that mango also possesses gastro-protective effects. To this purpose, Severi et al. [48] showed that a mango leaf decoction attenuated the gastric damage induced by HCl/ethanol in mice. This effect seems to be related to mangiferin and benzophenone glycoside, the main bioactive molecules present in leaf decoction. In this regard, Mahmoud-Awny et al. [49] reported that mangiferin mitigates gastric ulcer in ischemia/reperfused rats via inducing the expression of Nrf2, heme oxygenase and PPAR-γ (peroxisome proliferator-activated receptor gamma).

Bioactive compounds of mango have been also reported to exert anti-diabetic effects. Diabetes mellitus is a group of metabolic disorders associated with hyperglycaemia caused by defects in insulin secretion and/or action. Hyperglycaemia-induced advanced glycation end products (AGEs) activate their receptors (RAGEs) resulting in NF-κB-mediated release of pro-inflammatory cytokines. Activation of AGE-RAGE axis is associated with diabetic compliance, as cardiomyopathy and nephropathy. Mango mesocarp and leaf extracts produce a significant hypoglycaemic effect in streptozotocin (STZ)-induced diabetic rats [50,51,52]. Furthermore, Gondi et al. [53] showed that mango exocarp extracts also have the ability to ameliorate diabetes. In fact, administration of different doses of exocarp extracts to STZ-induced diabetic rats resulted in a significant decline in blood glucose levels, an increased plasma insulin level, as well as decreased levels of fructosamine and glycated haemoglobin, two diabetes status indicators. The anti-diabetic effect of mango exocarp extracts can be partially attributed to their ability to inhibit α-amylase and α-glucosidase, the carbohydrate hydrolysing enzymes. This effect may be due to the presence of polyphenolic acids, like gallic acid, chlorogenic acid, and ferulic acid, which have been shown to inhibit α-amylase and α-glucosidase activities [53].

## 5. Anticancer Effects of Mango

Bioactive components contained in the different parts of mango have also shown anticancer activity in different tumour cell lines. Nguyen et al. [54] showed that a methanol bark extract of *Mangifera indica* L. exerts cytotoxic effects in pancreatic cancer cells that correlated, among the isolated bioactive compounds, with mangiferolate and isoambolic acid. Ethanolic extract of mango exocarp induced apoptosis in human cervix adenocarcinoma HeLa cells by downregulating the anti-apoptotic factor Bcl-2 and activating caspase proteases [55]. This effect may be related to the presence of quercetin 3-*O*-galactoside, mangiferin gallate, isomangiferin gallate, quercetin-3-*O*-arabinopyranoside, and mangiferin. Furthemore, an aqueous extract of mango mesocarp has been reported to exert antitumor activity in a human colon adenocarcinoma cell line, as well as in a rodent model of colorectal cancer [56]. Abdullah et al. [57] reported that an ethanolic extract of mango kernel is able to induce cell death in both oestrogen-positive and -negative breast cancer cell lines, but not in normal breast cells. The cytotoxic effect of mango kernel extract in oestrogen-negative breast tumour cells has been correlated with the production of ROS, which promote apoptosis through Bax activation and cytochrome c release. In this context it has also been reported that gallic acid and gallotannin-rich mango extracts exert antitumor effects in BT474 breast cancer cells and athymic mice bearing BT474 cells as xenografts through suppression of the PI3K/Akt/mTOR pathway [58]. In addition, our recent results have demonstrated that Kensington Pride extracts prepared from Sicilian mango exocarp and compared with mango fruits from tropical areas can efficaciously exert an anti-proliferative action on human colon cancer cell lines (unpublished data). The data collected is very encouraging and suggests a targeted action on tumour cells. Our ongoing studies aim to explore the biochemical basis of this activity and characterise the phytochemical composition of Sicilian mango compared to the same cultivars grown in tropical areas and to the influence of the different ripeness grades.

Although only mesocarp is the edible fraction of the mango fruit, some cultures also used to eat the mango exocarp, probably knowing that the fruit peel contains a significant amount of healthful compounds that are present only in small amounts in the mesocarp. However, exocarp consumption can also promote an allergic reaction in some people because of either the presence of the oily organic allergen urushiol in the fruit peel [59], or the presence of pesticides used to counteract bacterial infections that can seriously affect plant life.

Therefore, these experimental observations open up the opportunity to isolate molecules active against cancer and opt for their use as nutritional supplements.

## 6. Mangiferin: An Unusual Natural Plant Polyphenol by Pleotropic Nutraceutical Features

Many studies, performed in order to analyse in details the chemical profiles and the mechanistic action of *Mangifera indica* fruit components, provided evidence that many of their anti-scavenging properties can be ascribed to mangiferin. Mangiferin is a plant natural polyphenol of xanthone structure with C-glucosyl linkage and four aromatic hydroxyl groups that have been considered crucial for its antiradical and antioxidant effect as well as for its pharmacological activity [60]. This polyphenolic xanthonoid is one of the most potent antioxidants known, mainly found in many *Anacardiaceae* and *Gentianaceae* plant families [61]. This molecule has also been highlighted in some medicinal herbs, influencing their therapeutic and preventive properties, and in honeybush (*Cyclopia* sp.), a popular herbal tea widely spread in South African areas. Mangiferin is highly soluble in water and can be easily extracted by infusion or in decoction preparations.

Mangiferin is differently distributed in many parts of mango plant and fruit. It has been found in the bark of plant (18.33 g/kg dry weight), in leaves and roots [62], in mesocarp, where its content can significantly vary depending on plant variety and fruit ripening stage (from 0.2 to 2.65 mg/kg dry weight) and in fruit exocarp (4.94 g/kg dry weight), which is the richest part of fruit in mangiferin [63].

The anti-free-radical action of mangiferin relies on its ability to directly neutralize ROS, such as hydroxyl radicals [64], superoxide anions, hydrogen peroxide [65], 2,2-diphenyl-1-picrylhydrazyl (DPPH), as well as in the scavenging property of lipid peroxides, peroxynitrite free radicals, and ROS induced by heavy metal exposure [60].

**Figure 2:**
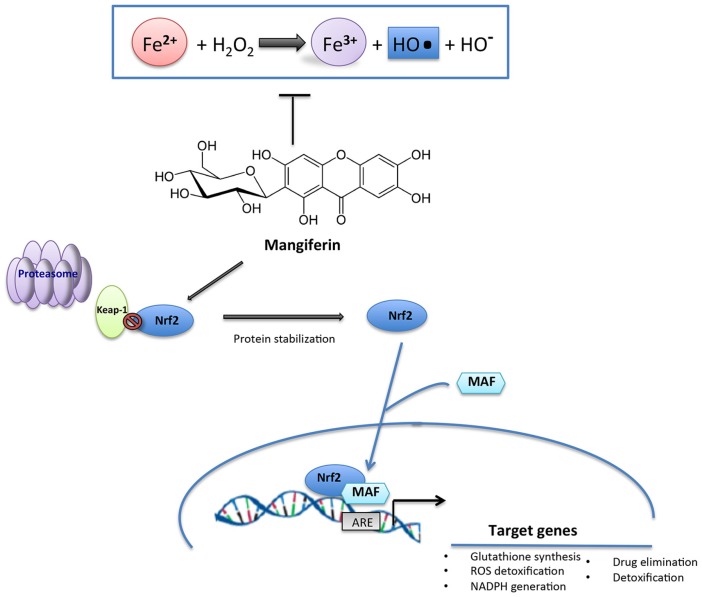
Schematic representation of the antioxidant actions of the xanthonoid mangiferin.

Compelling evidence demonstrated that mangiferin displays an efficient iron chelating potential (Figure 2), counteracting the hydroxyl radical generation in Fenton reaction [66]. Moreover, the anti-scavenging activity of mangiferin seems to be related to its ability to modulate the Nrf2/ARE (antioxidant response element) signalling detoxification pathway or promote the activation of key detoxifying enzymes [67]. Mangiferin has been proved to modulate Nrf2/ARE signalling pathway in healthy cells by increasing the Nrf2 half-life, the nuclear accumulation and the downstream production of NAD(P)H quinina reductase [67,68]. Many published studies also indicated mangiferin as a new promising anticancer bioactive compound [69] able to inhibit carcinogenesis and cancer cell growth by apoptosis induction both in vitro and in vivo systems [70]. It has also found application in cosmetics [71] due to both antioxidant and UV-protective action [72]. Evidence also shows that in a diabetic insulin-resistant rat model mangiferin caused a reduction of serum TNF-α and an elevation of serum adiponectin production as a consequence to PPAR-γ activation [73]. Moreover, mangiferin has also been shown to ameliorate diabetic compliances as cardiomyopathy and nephropathy. In this connection, Hou et al. [74] showed that, in a diabetic rat model, chronic treatment with mangiferin decreased the levels of myocardial enzymes and inflammatory mediators (TNF-α, IL-1β), as well as reduced the production of AGEs and their receptor RAGE.

Another study showed that chronic treatment with mangiferin significantly ameliorated renal dysfunction and reduced levels of AGEs and RAGEs in the renal cortex of diabetic rats. This last effect seems to be related to the ability of mangiferin to induce glyoxalase 1 (Glo-1), a detoxifying enzyme of methylglyoxal [75].

## 7. Vimang: The Cuban Mango Plant Extract with Antioxidant Potential and Beneficial Effects for Human Health

Beyond the nutraceutical activities observed with mango fruit extracts, a significant antiradical action has also been observed by other parts of the plant. An example of the nutraceutical potential of this plant is provided by an aqueous stem bark extract obtained from selected species of *Mangifera indica* L. that is used as nutritional supplement in Cuba [76] and commercialized under the brand name of Vimang^®^. When administered in animals, Vimang^®^ exhibited an anti-nociceptive and anti-inflammatory action against acetic acid exposure in mice [76]. Such an effect was attributed to the presence of micronutrients as selenium and different polyphenols such as phenolic acids, phenolic esters, flavan-3-ols, and mangiferin, the most abundant component that could account for its powerful scavenger activity [77,78]. This extract has been proved to be effective also against phospholipidic peroxidation in rat brains, counteracting DNA damage caused by iron/bleomycin or copper phenantroline exposure [79].

In some studies it has also been demonstrated that Vimang^®^ exerts a neuroprotective and cognitive enhancing action for mild cognitive impairment, a prodromal phase of dementia, by increasing the activity of some scavenger enzymes, such as superoxide dismutase, glutathione peroxidase, catalase, and lowering malondialdehyde levels [80].

## 8. Diffusion of *Mangifera indica* Cultivations in the Mediterranean Sicilian Area and its Impact on Sicilian Life

In the warm Mediterranean climate the main areas dedicated to the cultivation of *Mangifera indica* are the coastal regions of Portugal, Spain, Greece, Israel, and Italy. In particular, in Sicily, a Southern Italian island, many different provinces have been identified as areas particularly prone to the cultivation of subtropical fruits of mango (*Mangifera indica* L.), avocado (*Persea americana* Mill.), papaya (*Carica papaya* L.), and lychees (*Litchi chinensis* S.).

**Figure 3:**
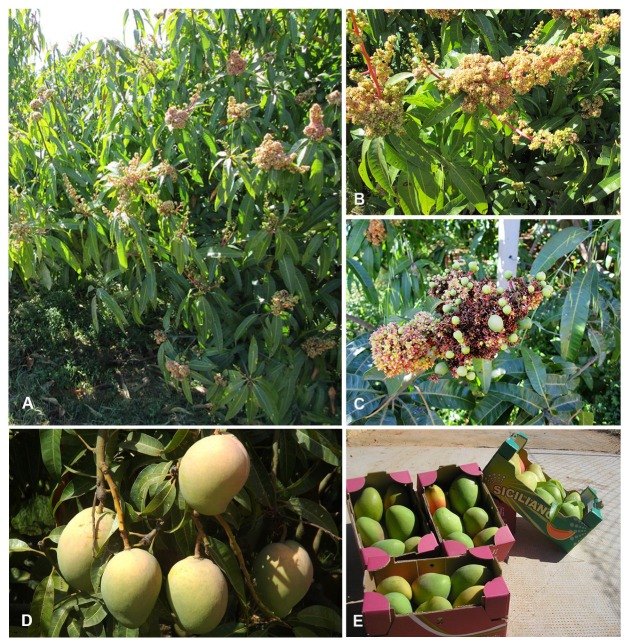
Sicilian mango plants and fruits. (**A**) Kensington Pride tree plantation in the province of Palermo (Balestrate). (**B**) Typical panicle inflorescence that stems at the apex of the branches carrying leaves and flowers. (**C**) Details of mango fruits growing on the inflorescence, the terminal shaped panicle. (**D**) Mango fruits of Kensington Pride variety from Balestrate cultivated land. (**E**) Sicilian mango fruits ready for the market. All pictures in the figure were provided by Ing. Luigi Martino.

Glenn, Maya, Tommy Atkins, Kensington Pride, and Keitt mango varieties spread out preferentially in Balestrate (Palermo), regions between Caronia and Milazzo (Messina), Acireale and Fiumefreddo (Catania) (Figure 3), where their introduction allowed the enhancement of the rural areas of Sicily, through a strategic pathway of growth and valorization. These subtropical cultivations are able to combine the needs of local productive realities and the requirements expressed by different consumers towards fruits endowed with nutritional and organoleptic properties. Fruits imported by Africa and Latin America to finally reach European market often lack flavour taste, scent, and bioactive components that are attractive to consumers. Differently, the Sicilian production is ideal for the fresh market and processing and can easily reach European consumers within 24–48 h.

The evaluation of organoleptic and sensory properties provided evidence that Sicilian mango fruit results of good quality. Analysing six different Sicilian cultivars, Farina et al. defined a sensory profile (UNI 10,957, 2003) based on the evaluation of 10 judges who randomly tested some sensory descriptors (colour, fibre, solidity, and exotic flavour) in 20 fruit samples for each mango variety [81].

**Figure 4:**
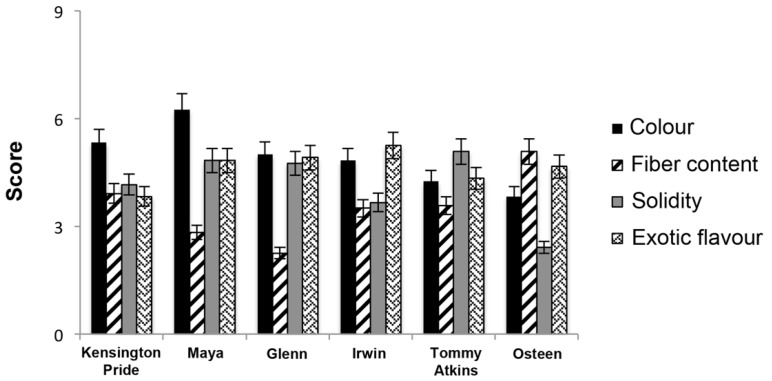
Sensory descriptors of six varieties of Sicilian mango. The panel evaluation was performed in a scale from 1 (extremely dislike) to 9 (extremely like). Data reported in the figure were extrapolated by previous studies performed by Farina et al. [81].

We summarized the analysis in Figure 4 showing that fibre content was high in the Osteen cultivar, while only an overall score of 2–3 was attained for the other Sicilian cultivars. The colour index for the mesocarp, usually considered as a good parameter of quality and perception of the consumer, was found higher for Maya (6.25) than Kensington Pride (5.33) and Glenn (5.00) cultivars.

Taken together, the organoleptic quality and sensory properties of local fresh and ripe mango fruit were considered better compared to those imported by extra-EU countries, such as China and Madagascar, since these fruits often arrive unripe to the consumers who do not appreciate them [82]. However, a well-defined analysis comparing fruits at the same grade of ripeness would be more appropriate to unequivocally define their profile.

We believe that a better understanding of the different healthy properties of this fruit could promote its consumption and favour Sicilian mango production. This could also have a strong impact on the Sicilian agricultural system enhancing marketplace requirements and creating new employment opportunities for people directly involved in the agricultural supply chain.

## 9. Conclusions

In modern society there is a growing interest in finding new bioactive molecules contained in the plants and fruits to be used both in the food and pharmaceutical industries. Fruits and vegetables are excellent sources of essential nutrients because of their high content in phytochemicals, such as phenolic compounds and flavonoids, which help to keep the consumer in good health. In particular, the present study summarised the most accurate evidence of the multifaceted actions of mango and its phytochemicals that have received a great deal of attention because of their beneficial potential in counteracting either the pro-inflammatory molecules or ROS production associated to human pathologies, such as cancer, cardiovascular diseases, aging, and neurodegenerative disorders.

The reported investigations were also compared to those known for Sicilian mango fruit. A better characterisation of phytocompounds found in Sicilian mango in comparison to those of tropical areas, as well as a broader analysis of their properties, could improve our knowledge of their biochemical activity and achieve the production of phytopharmaceuticals to associate with the most common therapies for some human disease treatments.

With regard to the bio-agronomic aspect, the acquisition of more information about mango’s potential, as well as the development of new supply chain strategies, could be of relevance for the Sicilian agricultural system.

## Figures and Tables

**Table 1:** Content of nutrients, vitamins, minerals, and carotenoids in *Mangifera indica* pulp.

*Mangifera indica* L. *Nutrition value per 100 g*	
Energy	60 Kcal
**Fruit composition**	**Quantity**
Carbohydrates	14.98 g
Protein	0.82 g
Fat	0.38 g
Fiber	1.6 g
**Vitamins**	
Vitamin C	36.4 mg
Vitamin E	1.12 mg
Vitamin A	1082 IU
Niacin (vit B3)	669 µg
Pantothenic acid (vit B5)	160 µg
Pyridoxine (vit B6)	119 µg
Riboflavin (vit B2)	38 µg
Thiamin (vit B1)	28 µg
Folates	43 µg
Vitamin K	4.2 µg
**Minerals**	
Potassium	168 mg
Phosphorus	14 mg
Calcium	11 mg
Magnesium	10 mg
Sodium	1 mg
Copper	110 µg
Iron	160 µg
Manganese	27 µg
Zinc	90 µg
**Carotenoids**	
β−Carotene	445 µg
α−Carotene	17 µg
